# Cytokine‐Induced Barrier Dysfunction and Lipid Signaling in a Gut‐On‐Chip Model

**DOI:** 10.1096/fj.202501685R

**Published:** 2025-10-09

**Authors:** Moran Morelli, Mariyana V. Savova, Karla Queiroz, Amy C. Harms, Thomas Hankemeier

**Affiliations:** ^1^ Mimetas Oegstgeest the Netherlands; ^2^ Metabolomics and Analytics Center, Leiden Academic Centre for Drug Research Leiden University Leiden the Netherlands

**Keywords:** cytokines, gut permeability, inflammation, intestinal integrity, intestine‐on‐chip, serum, signaling lipids

## Abstract

The intestinal epithelial barrier is crucial for gut homeostasis, with dysfunction linked to inflammatory disorders like inflammatory bowel disease (IBD). While cytokines are established mediators of barrier disruption, the role of lipid signaling remains poorly understood. Using a microfluidic platform, we cultured epithelial tubules and exposed their luminal (apical) side to TNF‐α, IL‐1β, and IFN‐γ (0–200 ng/mL) under serum‐containing or serum‐free conditions. Barrier function was assessed primarily via transepithelial electrical resistance (TEER), with DRAQ7 staining and actin cytoskeletal analysis providing complementary indicators of membrane integrity and structural disruption. In parallel, a targeted liquid chromatography–tandem mass spectrometry approach was used to profile lipid mediators across apical and basolateral compartments. Cytokine exposure significantly impaired barrier integrity, as indicated by reduced TEER, alongside associated cell damage and structural changes reflected by DRAQ7 staining and actin remodeling. The remodeling effect was lower in serum‐free medium. Lipid profiling revealed inflammatory signatures characterized by an increase in prostaglandins in the luminal compartment, particularly under serum‐free conditions. PGF1α increased under both media conditions, whereas the rest of the changes were condition‐specific, with a rise in PGE1, PGE2, and PGD2, among others, particularly under serum‐free conditions. Simultaneously, changes in other eicosanoids, but not in prostaglandins, were detected in the basolateral compartment under serum‐free conditions. This proof‐of‐principle study demonstrates how medium composition significantly influences inflammatory responses and lipid signaling patterns in a physiologically relevant gut‐on‐chip model. Our integrated approach reveals the complex spatial organization of lipid mediators during cytokine‐induced barrier dysfunction and provides a valuable framework for investigating the interplay between inflammation, barrier integrity, and lipid metabolism in intestinal pathophysiology.

## Graphical abstract

Using a microfluidic platform, this study evaluated how pro‐inflammatory cytokines affect intestinal barrier integrity and lipid signaling under both serum‐containing and serum‐free conditions. Exposure to a cytokine mixture compromised barrier function, increased cellular damage, and induced structural disruption. Notably, actin remodeling was less pronounced in serum‐free media. Lipidomic analysis revealed inflammatory profiles marked by elevated prostaglandin levels in the luminal compartment, especially under serum‐free conditions. Concurrently, changes in other eicosanoids were observed in the basolateral compartment in the absence of serum. [Created in BioRender. https://BioRender.com/bw96b7e]

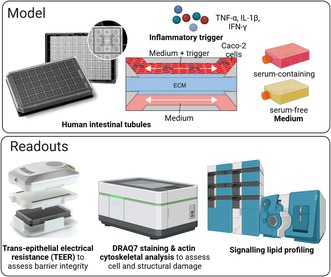

**Abbreviations**
CDCA: chenodeoxycholic acid
COX: cyclooxygenase
EMEM: Eagle's minimum essential medium
FBS: fetal bovine serum
HBSS: Hanks' balanced salt solution
IBD: inflammatory bowel disease
IFN‐γ: interferon‐gamma
IL‐1β: interleukin‐1 beta
LCA: lithocholic acid
LC–MS/MS: liquid chromatography–tandem mass spectrometry
MRM: multiple reaction monitoring
NF‐κB: nuclear factor kappa‐light‐chain‐enhancer of activated B cells
PBS: phosphate‐buffered saline
PG: prostaglandin
SF‐EMEM: serum‐free Eagle's minimum essential medium
TDCA: taurodeoxycholic acid
TEER: transepithelial electrical resistance
TNF‐α: tumor necrosis factor‐alpha

## 1 Introduction

The intestinal epithelial barrier is crucial for maintaining health. It facilitates nutrient absorption, supports immune defense, and prevents pathogen invasion [1, 2]. It also regulates the selective permeability of the gut, allowing essential nutrients to pass into the bloodstream while blocking harmful substances. Disruption of this barrier is associated with various inflammatory and metabolic disorders, including inflammatory bowel disease (IBD) and food allergies [3, 4]. Understanding the mechanisms that preserve gut barrier integrity is essential for developing therapeutic strategies for these conditions.

Cytokines play a central role in the disruption of intestinal barrier integrity. Elevated levels of cytokines such as tumor necrosis factor‐alpha (TNF‐α), interleukin‐1 beta (IL‐1β), and interferon‐gamma (IFN‐γ) contribute to barrier dysfunction by disrupting tight junction integrity and increasing epithelial permeability. For instance, TNF‐α and IFN‐γ have been shown to synergistically impair barrier function by altering tight junction protein expression and distribution [5, 6]. Additionally, IL‐1β can induce barrier dysfunction by promoting inflammatory responses that compromise epithelial cell integrity [7]. These cytokines also induce actin cytoskeletal remodeling [8], which is directly linked to compromised epithelial barrier function and increased permeability [9].

While the role of cytokines in intestinal barrier dysfunction is well‐documented, the role of other inflammatory mediators remains understudied. Eicosanoids, potent bioactive signaling lipids derived from arachidonic acid and eicosapentaenoic acid, may also play a role in maintaining intestinal barrier integrity [10]. Among them, prostaglandins (PG), more specifically increased levels of prostaglandin E2 (PGE2), have been associated with increased gut permeability in in vitro models [11, 12], whereas elevated mucosal, fecal, and blood levels of eicosanoids have been reported in patients with ulcerative colitis [13, 14]. Despite this, the role of eicosanoids and other signaling lipids in barrier regulation and inflammatory responses remains poorly understood.

To better understand lipid signaling pathways and their role in barrier regulation, appropriate experimental models are essential. However, traditional in vitro models such as static cell culture often fail to replicate the complex architecture and dynamic environment of the intestinal epithelium, limiting their relevance for studying gut physiology and pathology [15]. Advanced models, such as organ‐on‐chip systems, overcome these limitations by incorporating microfluidic technology to mimic the mechanical forces, spatial organization, and cell–cell interactions of the intestine [16, 17]. The OrganoPlate, a membrane‐free microfluidic platform, offers a robust solution by allowing the culture of up to 64 intestinal tubules with apical and basolateral access [18]. It provides simultaneous assessment of barrier function and cellular responses, making it ideal for complex analyses combining barrier permeability [19, 20, 21] and inflammation [22, 23].

While these advanced models provide powerful tools for studying intestinal biology, optimizing their experimental conditions is crucial for reliable results. Particularly, the composition of cell culture media is known to significantly influence cellular responses to external stimuli [24]. Serum starvation is a common practice in drug screening, primarily used to synchronize the cell cycle [25]. However, it has limitations, as prolonged starvation can have undesirable effects on cell viability and function [26], and cells cannot be maintained in a starved state indefinitely. Conversely, serum provides essential nutrients, binds and transports small molecules including lipids [27], and reduces non‐specific binding to culture plastic surfaces [28], but it introduces batch variability and is a possible source of contamination [29]. The impact of serum on signaling lipid secretion in intestinal barrier models remains underexplored. This gap highlights the need for systematic studies to evaluate how medium composition affects cytokine‐induced barrier dysfunction and lipid signaling.

Here, we aim to address these gaps using the OrganoPlate to investigate the effects of cytokine exposure on intestinal barrier integrity and signaling lipid secretion under varying medium conditions. Specifically, we compare standard serum‐containing and serum‐free media to evaluate their influence on cytokine‐induced changes in barrier function, assessed through transepithelial electrical resistance (TEER), DRAQ7 staining (as a marker of cell death), actin remodeling, and lipid signaling. By focusing on the interplay between cytokine responses and medium composition, this study provides a novel framework for understanding the mechanisms underlying gut inflammation.

This proof‐of‐principle study represents a significant advancement in intestinal research, offering a comprehensive analysis of cytokine‐induced barrier dysfunction alongside lipid signaling in a microfluidic platform. By leveraging the OrganoPlate technology, we demonstrate how physiologically relevant models can uncover previously unexamined dynamics of gut inflammation, paving the way for innovative experimental designs and therapeutic strategies.

## 2 Materials and Methods

A detailed list of all chemicals and reagents is provided in the Supporting Information.

### 2.1 OrganoReady Colon Caco‐2

**FIGURE 1:**
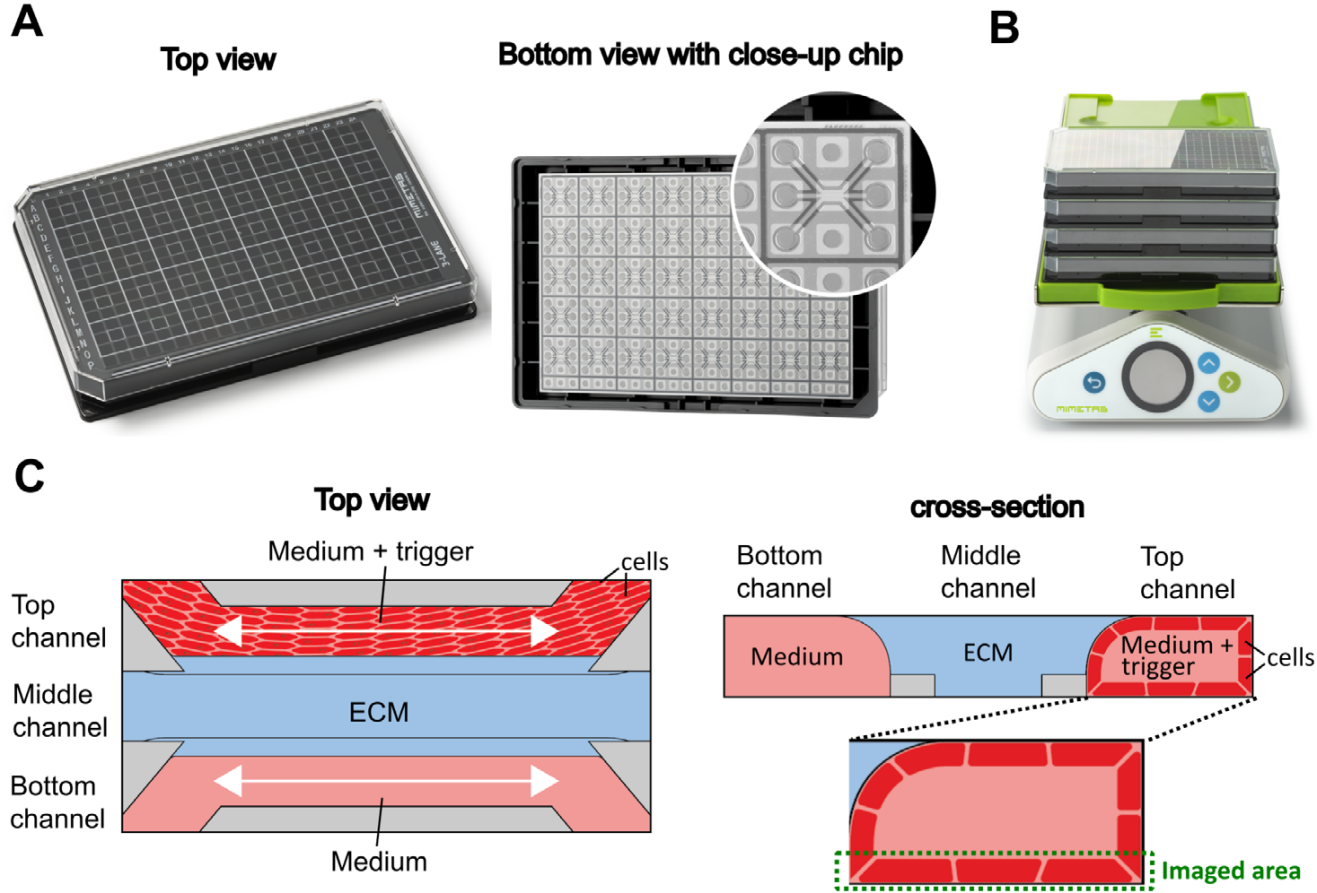
Overview of the OrganoPlate platform and experimental configuration. (A) Illustration of the OrganoPlate showing top and bottom view with a close‐up on a microfluidic chip. (B) OrganoFlow rocker generating pump‐free medium perfusion. (C) Detailed schematic of the three‐channel structure (top and cross‐sectional views), highlighting the top channel where the epithelial cell tubule is cultured. This top channel represents the luminal (apical) side of the tubule, where cytokines are administered. The middle channel contains ECM, and the bottom channel contains culture medium and represents the basolateral side of the tubule. The dashed green outline indicates the imaged area at the bottom of the tubule used for maximum projection analysis.

The OrganoReady Colon Caco‐2 3‐lane 40 (MI‐OR‐CC‐01, MIMETAS B.V.) cultures are pre‐seeded Caco‐2 tubules in the OrganoPlate platform—a multiwell plate format equipped with microfluidic chips designed to support up to 64 membrane‐free microfluidic chips. Following Trietsch et al. [18], these cultures were prepared according to the manufacturer's instructions, with Caco‐2 medium replaced upon receipt. Continuous perfusion flow was applied using the OrganoFlow rocker (MIMETAS B.V., MI‐OFPR‐L), set at a 7‐degree angle with 8‐min intervals optimized specifically for the 3‐lane 40 configuration. On day 2 post‐receipt (day 6 post‐seeding), the medium was refreshed, and experimental exposures began on day 4 post‐receipt (day 8 post‐seeding). See Figure 1 for a visual representation of the OrganoPlate and OrganoFlow setup.

### 2.2 Cytokines Exposures and Sampling

On day 8 post‐seeding, Caco‐2 tubules in the OrganoPlate were exposed to a cytokine mixture. The culture medium in the bottom and middle channels was replaced with complete Eagle's Minimum Essential Medium (EMEM) or serum‐free EMEM (SF‐EMEM). For the top channel, the medium was replaced with either EMEM or serum‐free EMEM containing TNF‐α, IL‐1β, and IFN‐γ at concentrations of 50, 100, or 200 ng/mL each. The plates were placed back in the incubator and maintained under continuous perfusion.

After 72 h of exposure and endpoint readouts, culture medium was collected from the top, middle, and bottom channels. All experiments were performed three times independently (*n* = 3), and each independent experiment contained four chips per condition (*n* = 4). For the cellular assays, each replicate was considered during the statistical analysis. For the signaling lipid profiling, per condition and channel, media from four replicate chips (~80 μL each) were pooled to prepare a sample, resulting in three independent replicates per condition (*n* = 3). The (pooled) samples were centrifuged for 10 min at 1500 rpm to remove dead cells. Media samples and media spiked with 200 ng/mL cytokine mixture were collected for background assessment. All samples were stored at −80°C immediately after collection. A close‐up of the chip configuration and different channels (top, middle, bottom) is shown in Figure 1C.

### 2.3 Trans‐Epithelial‐Electrical‐Resistance (TEER)

**FIGURE 2:**
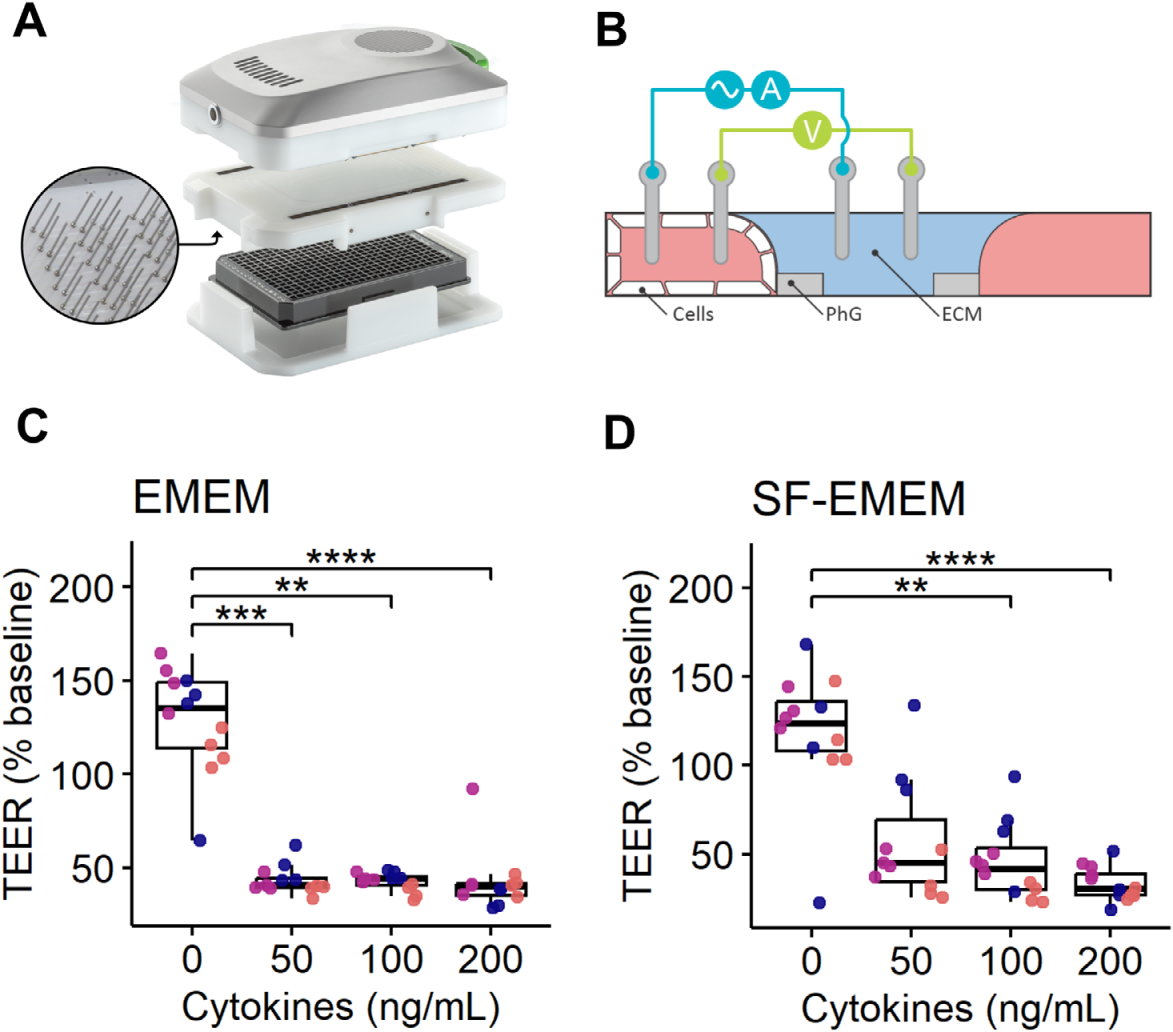
Impact of Cytokine exposure on TEER in complete (EMEM) and serum‐free (SF‐EMEM) media. (A) OrganoTEER setup to measure TEER in an OrganoPlate. (B) Schematic diagram showing a transversal view of the OrganoPlate chip and the electrode pair positioning across the medium channel and the Caco‐2 tube channel containing the medium dilution with cytokine (TNF‐α, IL‐1β, and IFN‐γ). The concentration is expressed as ng/nl of each cytokine. PhG: PhaseGuide, ECM: ExtraCelluar Matrix. (C‐D) Dose‐dependent decrease in TEER after exposure to cytokines in EMEM (C), and SF‐EMEM (D). Data represent the percentage change in TEER compared to baseline measurements. Each dot represents a measurement from a single chip in the OrganoPlate. Colors indicate independent experiments. Results are derived from three independent experiments (*N* = 3), with four technical replicates per cytokine concentration (*n* = 4). Significance was determined using Kruskal–Wallis test followed by Dunn's post hoc test, with *p* values adjusted using the Bonferroni correction. Adjusted *p* values are expressed as follows: ****p < 0.0001, ***p < 0.001, **p < 0.01.

To evaluate the integrity of the gut barrier, TEER was measured with the OrganoTEER, an automated multichannel impedance meter [20]. An electrode board, designed to fit the 3‐lane OrganoPlate, was sterilized with 70% ethanol for at least 1 h before measurement. The OrganoPlate was taken out of the incubator and equilibrated at room temperature for 30 min before the measurement to eliminate any flow or temperature effect. Baseline measurements were performed on day 8, after equilibration and right before exposure, then TEER was further measured after 72 h of exposure to the cytokine mixture. For Caco‐2, the OrganoTEER was set on “high TEER” and we used a TEER threshold of 350 Ω/cm^2^ at baseline, meaning that all tubules below 350 Ω/cm^2^ at baseline were excluded from the analysis. The OrganoTEER setup is shown in Figure 2A,B.

### 2.4 Fluorescent Imaging

To visualize the effect of cytokine exposure on cell structure and membrane integrity, the tubules were directly fixed and stained on the OrganoPlate.

#### 2.4.1 DRAQ7 Nuclear Staining

Before fixation, culture medium in the tubule inlets and outlets was replaced by 20 μL of DRAQ7 dye (Biostatus, DR71000) diluted 1:100 in serum‐free EMEM, and cells were incubated for 30 min under continuous perfusion inside the incubator.

#### 2.4.2 Fixation

Intestinal tubules were fixed with 3.7% formaldehyde (Sigma, 252 549) in Hanks' balanced salt solution (HBSS) with calcium and magnesium (Thermo Scientific, 14 025 092) for 15 min, washed twice with phosphate‐buffered saline (PBS; Gibco, 70 013 065) for 5 min, and then stored with 50 μL PBS per well at 4°C until further staining.

#### 2.4.3 Actin and Hoechst Nuclear Staining

Intestinal tubule cells were permeabilized with 0.03% Triton X‐100 (Sigma, T8787) in PBS for 10 min, followed by two washes with 4% FBS in PBS. Cells were stained with NucBlue Fixed Cell ReadyProbes Reagent (Invitrogen, R37606) and ActinGreen (Invitrogen, R37110) as per the manufacturer's instructions. Two drops per ml of both NucBlue and ActinGreen were added to PBS to prepare the staining solution. Twenty microliters of the staining solution was introduced at both the inlet and outlet of the tube, allowing for incubation at room temperature for 30 min under continuous perfusion. Cells were then washed twice with PBS for 5 min each.

### 2.5 Intestinal Tubules Visualization and Imaging

Imaging was conducted on the bottom of the tubules using the ImageXpress Micro XLS (Molecular Devices, USA) and Micro XLS‐C High Content Imaging Systems (Molecular Devices, USA) and processed using Fiji34 to enhance contrast and improve visualization. Fixed and stained OrganoPlates were stored at 4°C until imaging and equilibrated at room temperature for at least 30 min before imaging. Maximum intensity projection images were saved as TIFF files after confocal imaging of stained cells.

### 2.6 Quantification of DRAQ7 and Actin Signals

We used the open‐source cell image analysis software CellProfiler (version 4.2.5) to process the images. We designed a pipeline to process the TIFF files capturing DRAQ7 and NucBlue staining, allowing the segmentation of DRAQ7‐positive cells and total nuclei to quantify the percentage of DRAQ7‐positive (DRAQ7 +) cells. Another pipeline was designed to quantify actin remodeling. We identified actin objects from the actin signal and analyzed three parameters: actin object count, object mean area, and total actin area, which together quantify the number, individual size, and total extent of actin reorganization. All parameters were divided by nuclei count (NucBlue staining) to normalize for cell number. Detailed methodology of the staining and CellProfiler pipeline was previously described [30].

### 2.7 Signaling Lipids Profiling

#### 2.7.1 Sample Collection and Preparation

An aliquot of 150 μL of pooled media was subjected to liquid–liquid extraction as described by Yang et al. (2024) with small modifications [31]. Briefly, to 150 μL of media, 5 μL antioxidant solution (0.2 mg mL^‐1^ butylated hydroxytoluene (BHT) and 0.2 mg mL^‐1^ ethylenediaminetetraacetic acid (EDTA)), 10 μL of internal standard solution, 150 μL of citrate buffer (0.2 M citric acid, 0.4 M disodium hydrogen phosphate buffer, pH 4.5) and 1 mL of the butanol:methyl *tert*‐butyl ether (BuOH:MTBE) (1:1 v:v) were added. The samples were left on ice for 20 min, followed by homogenization for 4 min in a Bullet Blender. Following centrifugation at 15 800 rcf (10 min, 4°C), 900 μL of the upper organic phase was collected and dried under vacuum in Speedvac (Labcono, USA). The samples were reconstituted in 40 μL of methanol/acetonitrile (7:3 v:v), vortexed for 5 min, centrifuged at 15800 rcf (10 min, 4°C), and transferred to injection vials for liquid chromatography with tandem mass spectrometry (LC–MS/MS) analysis.

#### 2.7.2 LC–MS/MS Conditions

LC–MS/MS analysis, covering a range of isoprostanes, prostaglandins, endocannabinoids, and bile acids, was carried out as previously described [31]. Briefly, the analysis was carried out on a three‐pump Shimadzu LC‐30 AD system coupled to a SCIEX Triple‐Quad 7500 system (USA). The chromatographic separation was performed on a Waters Acquity BEH C18 column (50 mm × 2.1 mm, 1.7 μm) at 40°C following a 16‐min multistep gradient at a flow rate of 0.7 mL/min. Mobile phase A consisted of 0.1% (v/v) acetic acid in water, mobile phase B of 0.1% acetic acid in acetonitrile/methanol (90/10, v/v), and mobile phase C of 0.1% acetic acid in isopropanol. The data were acquired under multiple reaction monitoring (MRM) at unit resolution with polarity switch scanning mode. A detailed list of MRM transitions and retention times of each analyte and the corresponding isotopically labeled standard can be found in the original work [31]. Data acquisition of the main and follow‐up experiments was carried out on SCIEX OS 2.0.0 and SCIEX OS 3.3.0.

#### 2.7.3 Quality Control

Blank media samples (EMEM and SF‐EMEM) were used to assess the background signal from each media type. In a follow‐up experiment, the background signal coming from the cytokine mixture was assessed by analyzing blank media samples (EMEM and SF‐EMEM) and media (EMEM and SF‐EMEM) spiked with 200 ng/mL cytokine mixture.

### 2.8 Data Analysis

The statistical analyses for cellular assays (TEER measurement, actin remodeling, and DRAQ7 signal) were conducted in two steps. First, we performed a global analysis of main effects and interactions on the full dataset using Two‐Way ANOVA when data met normality assumptions (verified by Shapiro–Wilk test *n* > 50, or Anderson‐Darling test *n* > 50) or Scheirer–Ray–Hare test for non‐normal distributions. This analysis evaluated the effects of cytokine cocktail concentration (0, 50, 100, 200 ng/mL) and medium type (EMEM vs. SF‐EMEM) on cellular assays, as well as the interaction between both variables.

For variables showing significant main effects, we conducted independent analyses. When comparing cytokine concentrations, we used One‐Way ANOVA followed by Tukey's HSD post hoc test for normal data, or Kruskal–Wallis followed by Dunn's post hoc test for non‐normal data. When medium type comparisons were significant, we used an independent t‐test for normal data or a Wilcoxon Rank‐Sum test for non‐normal data. All *p* values were adjusted for multiple comparisons using Bonferroni correction, with adjusted values (*q* values) ≤ 0.05 considered statistically significant. Global analysis results and detailed analyses of the medium are provided in Supporting Information.

Preprocessing of the LC–MS/MS data was performed using SCIEX OS 3.0. Data analysis and visualization were conducted using R version 4.3.2. Per media, lipids with fewer than three measurements below three times the blank media signal (blank threshold) were excluded. Data were visually inspected, and only lipids detected above the blank threshold in at least two conditions (defined by media, channel, and concentration) were retained. The cytokine mixture was evaluated for potential background interference, and only lipids unaffected by it were retained.

## 3 Results

### 3.1 Cytokine Mixture Significantly Reduced TEER in EMEM and SF‐EMEM Medium Conditions

TEER measurements reflect epithelial barrier integrity, with decreases indicating increased permeability, characteristic of inflammation‐driven barrier dysfunction. We exposed Caco‐2 tubules to a cytokine mixture (TNFα, IL‐1β, and IFNγ) at concentrations of 0, 50, 100, and 200 ng/mL each for 72 h in two different media (EMEM and SF‐EMEM). Using OrganoTEER technology (Figure 2A,B), we assessed barrier integrity through real‐time, non‐invasive TEER measurements before and after exposure. Values were normalized to baseline (i.e., expressed as a percentage of the chip's pre‐exposure value) and analyzed to evaluate cytokine effects in EMEM and SF‐EMEM.

The Scheirer–Ray–Hare test revealed a significant effect of cytokine concentration on TEER (*p* = 7.0 × 10^−7^) but an insignificant effect of medium type (*p* = 0.73) and the cytokine–medium interaction (*p* = 0.95) (Table S1). In EMEM, all cytokine concentrations (50, 100, and 200 ng/mL) significantly reduced TEER compared to control, with no differences between treatment groups (Figure 2C). In SF‐EMEM, only higher concentrations (100 and 200 ng/mL) significantly reduced TEER versus control (Figure 2D).

### 3.2 Cytokine Mixture Increased DRAQ7 Signal in All Conditions Independently of Media Composition

**FIGURE 3:**
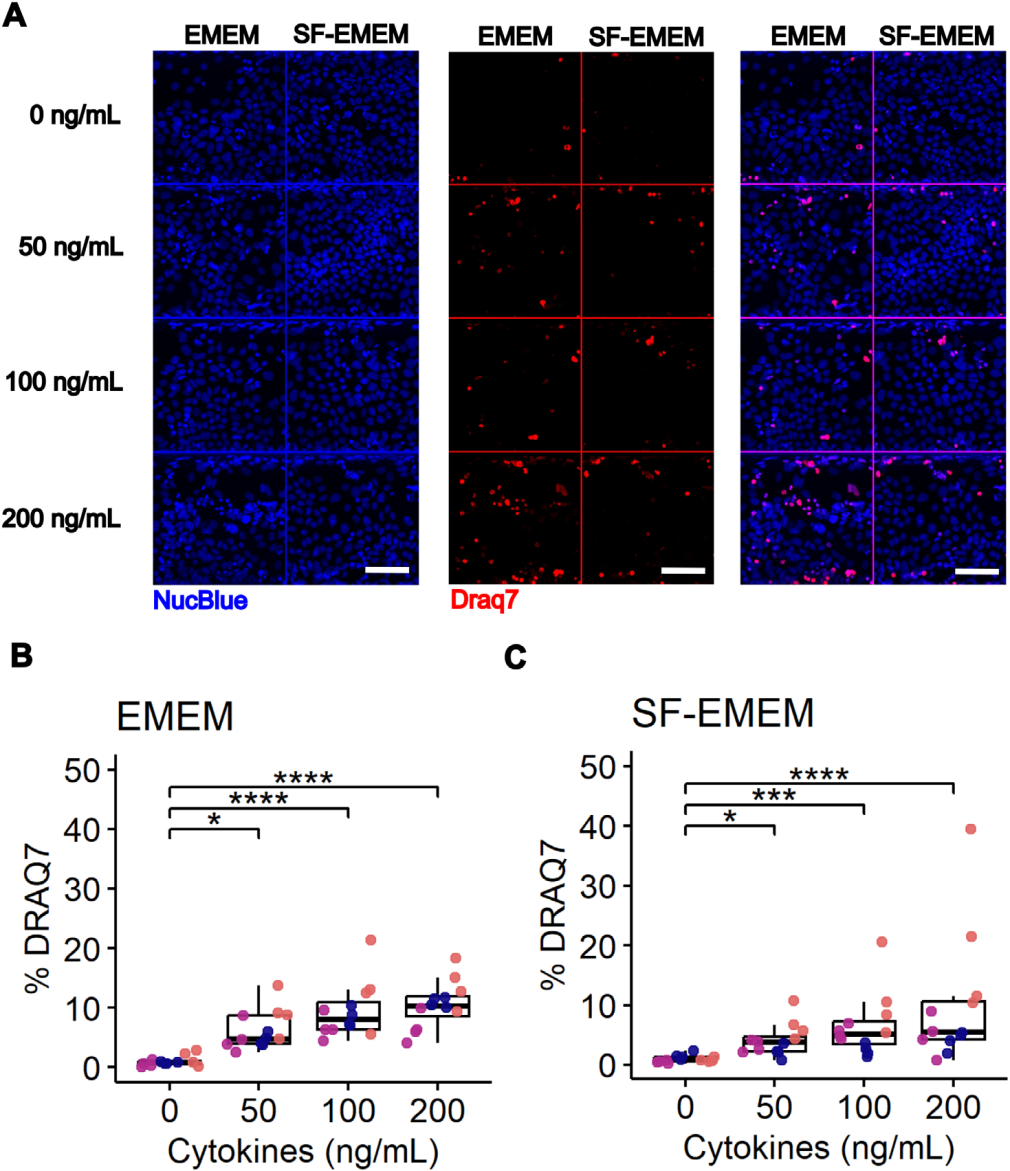
Impact of cytokine exposure on Cmaco‐2 tubule membrane integrity to DRAQ7 in EMEM and SF‐EMEM media. (A) Representative fluorescence images of NucBlue (blue, nuclei) and DRAQ7 (red, dead cells) under various cytokine conditions. Scale bar = 100 μm. (B‐C) Quantification of DRAQ7‐positive cells (% DRAQ7) in Caco‐2 tubules exposed to increasing concentrations of cytokines (TNF‐α, IL‐1β, and IFN‐γ) in EMEM (B) and SF‐EMEM (C). The concentration is expressed as ng/nl of each cytokine. Data represent the percentage of DRAQ7‐positive cells normalized to total nuclei count. Each dot represents a measurement from a single chip in the OrganoPlate. Colors indicate independent experiments. Results are derived from three independent experiments (*N* = 3), with four technical replicates per cytokine concentration (*n* = 4). Statistical significance was determined using the Kruskal–Wallis test followed by Dunn's post hoc test, with *p* values adjusted using the Bonferroni correction. Adjusted *p* values are expressed as follows: ****p < 0.0001, ***p < 0.001, *p < 0.05.

The effect of cytokine exposure on Caco‐2 tubule integrity was assessed using DRAQ7 staining (Figure 3A). DRAQ7 is a membrane‐impermeant DNA dye that labels nuclei of cells with compromised plasma membranes. It is therefore commonly used as a marker of cell death. To quantify this effect, we performed DRAQ7 staining and developed a CellProfiler pipeline to analyze the percentage of DRAQ7‐positive nuclei.

The Scheirer‐Ray‐Hare test showed a significant effect of cytokine concentration (*p* = 1.05 × 10^−11^) and medium (*p* = 0.033) on DRAQ7 positive‐nuclei with no significant interaction between the two variables (*p* = 0.62) (Table S2). DRAQ7 signal increased significantly when cytokine concentrations were raised from 0 (control) to each dose (50, 100, or 200 ng/mL) in both media types. However, no significant differences were observed between the doses themselves (e.g., between 50 and 100, or 100 and 200 ng/mL) (Figure 3B,C). Post hoc comparisons did not reveal significant differences between media at each cytokine concentration (Table S2).

### 3.3 Cytokine Mixture Induced Actin Remodeling in All Conditions After Exposure

**FIGURE 4:**
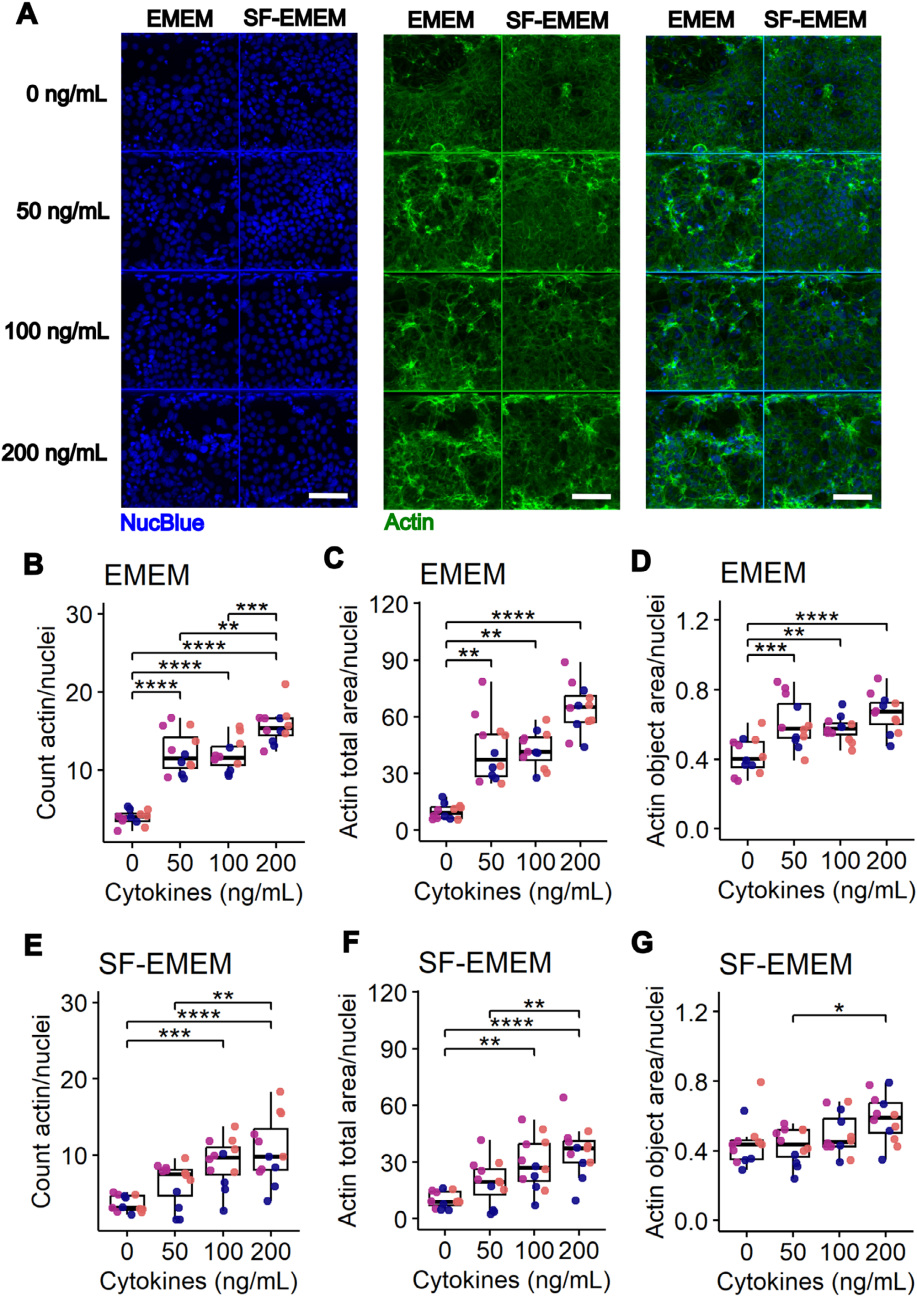
Impact of cytokine exposure on Actin remodeling in EMEM and SF‐EMEM media. (A) Representative fluorescence images of NucBlue (blue, nuclei) and Actin (green) under various cytokine conditions. Scale bar = 100 μm. (B‐G) Quantification of Actin remodeling parameters, including the count of Actin objects per nucleus (B, E), total Actin area per nucleus (C, F), and mean Actin object area per nucleus (D, G), in Caco‐2 tubules exposed to increasing concentrations of cytokines (TNF‐α, IL‐1β, and IFN‐γ) in EMEM (B‐D) and SF‐EMEM (E‐G). The concentration is expressed as ng/nl of each cytokine. Each dot represents a measurement from a single chip in the OrganoPlate. Colors indicate independent experiments. Results are derived from three independent experiments (*N* = 3), with four technical replicates per cytokine concentration (*n* = 4). Statistical significance was determined using either the Kruskal–Wallis test followed by Dunn's post hoc test or ANOVA followed by Tukey's post hoc test, depending on whether assumptions for parametric tests were met, with *p* values adjusted using the Bonferroni correction. Adjusted *p* values are expressed as follows: ****P < 0.0001, ***p < 0.001, **p < 0.01, *p < 0.05.

The effect of cytokine exposure on actin remodeling was assessed using Actin staining (Figure 4A). Actin remodeling reflects cytoskeletal reorganization, which can occur in response to environmental stressors, such as cytokine exposure, and may serve as an early indicator of barrier disruption and epithelial dysfunction under inflammatory conditions.

#### 3.3.1 Actin Count

Two‐Way ANOVA revealed significant effects of cytokine concentration (*p* = 4.17 × 10^−20^), medium type (*p* = 2.98 × 10^−9^), and their interaction (*p* = 0.0019) on actin object count, indicating dependent effects of cytokine mixture and medium (Table S3). In EMEM, all cytokine concentrations increased actin count versus control, with 200 ng/mL showing higher counts than 50 or 100 ng/mL (Figure 4B). In SF‐EMEM, increases were significant only at 100 and 200 ng/mL versus control, with 200 ng/mL also higher than 50 ng/mL (Figure 4E). Medium‐specific comparisons revealed significantly lower actin counts in SF‐EMEM compared to EMEM at 50 and 200 ng/mL, but not at 0 or 100 ng/mL (Figure S1).

#### 3.3.2 Total Actin Area

The Scheirer–Ray–Hare test showed significant effects of cytokine concentration (*p* = 5.19 × 10^−11^) and medium type (*p* = 3.49 × 10^−4^) on actin total area, while the cytokine–medium interaction was not significant (*p* = 0.14), indicating independent effects of cytokine mixture and medium (Table S4). In EMEM, all cytokine concentrations increased the total actin area compared to control, with no differences between treated groups (Figure 4C). In SF‐EMEM, increases were significant at 100 and 200 ng/mL versus control and at 200 ng/mL versus 50 ng/mL (Figure 4F). Medium comparisons within each cytokine condition showed significantly lower actin area in SF‐EMEM compared to EMEM at 50, 100, and 200 ng/mL, but not at 0 ng/mL (Figure S2).

#### 3.3.3 Mean Actin Object Area

Two‐Way ANOVA showed significant effects of cytokine concentration (*p* = 6.11 × 10^−6^), medium type (*p* = 0.00175), and their interaction (*p* = 0.0266), indicating dependent effects of cytokine mixture and medium on actin object area (Table S5). In EMEM, only 200 ng/mL significantly increased the mean object area versus control (Figure 4D). In SF‐EMEM, cytokine exposure showed no significant effects (Figure 4G). Medium comparisons revealed a significantly lower mean object area in SF‐EMEM compared to EMEM only at 50 ng/mL (Figure S3).

### 3.4 Lipid Signaling Profiling

#### 3.4.1 Cytokine Exposure Resulted in Higher Prostaglandin Levels in the Top Channel Under SF‐EMEM Conditions

The effect that cytokine exposure has on the signaling lipid profile in Caco‐2 cells was assessed by examining the changes to a range of isoprostanes, prostaglandins, endocannabinoids, and bile acids. Under the SF‐EMEM and EMEM conditions, respectively, 40 of 105 and nine of 125 detected signaling lipids met the quality control criteria and were studied further (Table S6).

**FIGURE 5:**
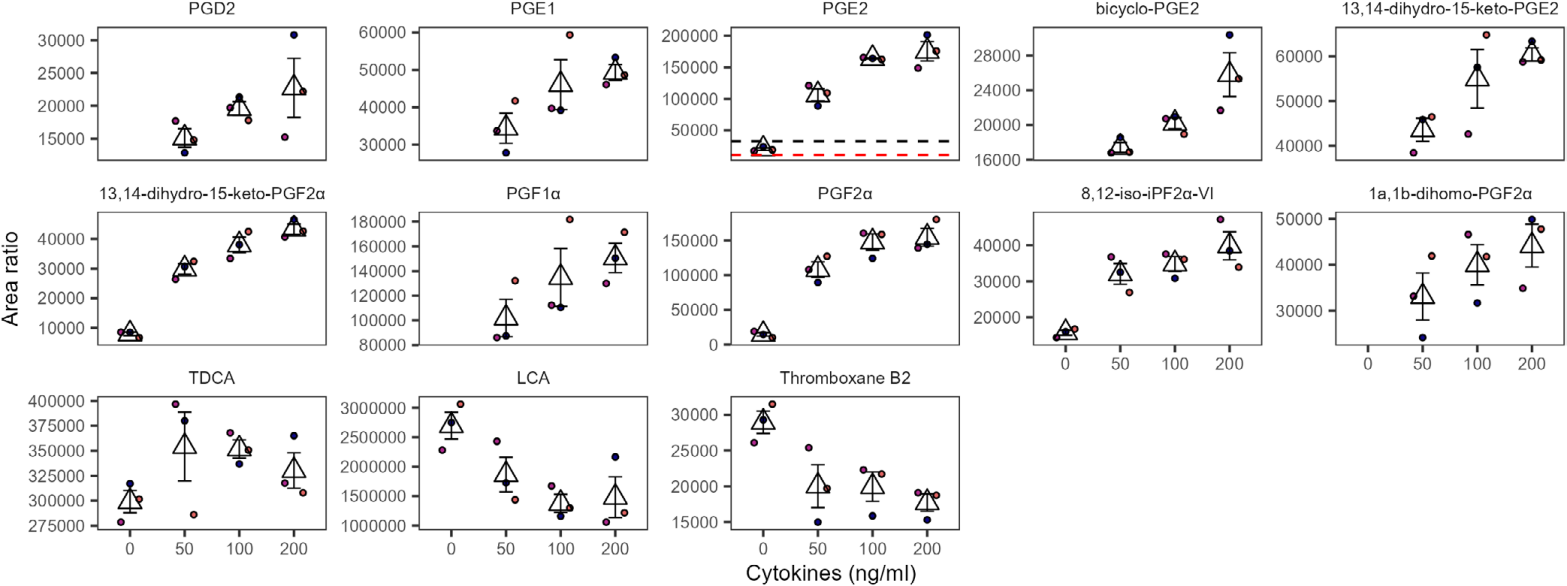
Impact of cytokine exposure on signaling lipid in SF‐EMEM media, top channel. Results are derived from three independent experiments (*N* = 3). Missing measurement points correspond to signals below the detection limit. Dot colors indicate independent experiments. The triangular marker represents the mean of the three replicates, while the error bars are based on the standard deviation. The red and black dotted lines represent, respectively, one and three times the median level in blank SF‐EMEM. The results for PGF2α, 8,12‐iso‐iPF2α‐VI, and 13,14‐dihydro‐15‐keto‐PGE2 should be treated with caution since EMEM media spiked with cytokine mixture contributed to the background signal of those lipids. The concentration is expressed as ng/mL of each cytokine.

Under the SF‐EMEM conditions, 12 signaling lipids were affected by the cytokine exposure in the top channel (Figure 5). Among those, nine prostaglandins (PGs) and derivatives, and the isoprostane 8,12‐iso‐iPF2α‐VI were secreted in response to the cytokine exposure in a concentration‐dependent manner. The cytokine exposure also affected the bile acids taurodeoxycholic acid (TDCA) and lithocholic acid (LCA), and thromboxane B2. The levels of the latter two declined upon exposure to the cytokine cocktail, which contrasted with the other observed changes.

**FIGURE 6:**
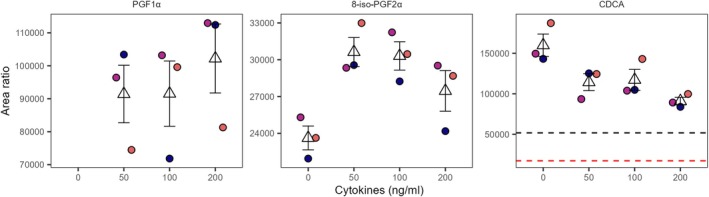
Impact of cytokine exposure on selected signaling lipid in EMEM media, top channel. Results are derived from three independent experiments (*N* = 3). Missing measurement points correspond to signals below the detection limit. Dot colors indicate independent experiments. The triangular marker represents the mean of the three replicates, while the error bars are based on the standard deviation. The red and black dotted lines represent, respectively, one and three times the median level in blank EMEM. The concentration is expressed as ng/mL of each cytokine.

Under EMEM conditions, PGF1α, 8‐iso‐PGF2α, and chenodeoxycholic acid (CDCA) were affected by the cytokine exposure in the top channel (Figure 6). Analogous to the SF‐EMEM results, PGF1α was higher in the cytokine‐exposed conditions compared to the unexposed conditions. Relative to the baseline, 8‐iso‐PGF2α was also higher when the tubules were exposed to 50 and 100 ng/mL of cytokine mixture. Other PGs and derivatives, for example, PGE2 and bicyclo‐PGE2, were also higher in the cytokine‐exposed conditions compared to the control; however, they did not meet our quality criteria due to the high EMEM or cytokine mixture background (Figure S4, Table S6). The primary bile acid CDCA, in contrast, declined with the addition of cytokines to the media (Figure 6).

#### 3.4.2 Concentration‐Dependent Decline in Eicosanoids After Exposure to Cytokines in the Bottom Channel Under SF‐EMEM Conditions

**FIGURE 7:**
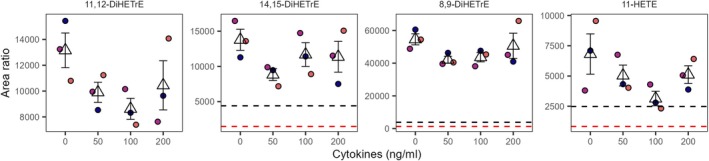
Impact of cytokine exposure on signaling lipid profiles in SF‐EMEM media, bottom channel. Results are derived from three independent experiments (*N* = 3). Dot colors indicate independent experiments. The triangular marker represents the mean of the three replicates, while the error bars are based on the standard deviation. The red and black dotted lines represent, respectively, one and three times the median level in blank SF‐EMEM. The concentration is expressed as ng/mL of each cytokine.

None of the signaling lipids that changed with the cytokine exposure in the top channel exhibited a similar effect in the bottom channel. Instead, in the bottom channel under the SF‐EMEM conditions, the eicosanoids 8,9‐DiHETrE, 11,12‐DiHETrE, 14,15‐DiHETrE, and 11‐HETE were affected in a cytokine concentration‐dependent manner (Figure 7). For the 8,9‐DiHETrE and 11,12‐DiHETrE, the decline was evident at 50 ng/mL and 100 ng/mL of cytokines, whereas for 14,15‐DiHETrE, only at the 50 ng/mL level (Figure 7). Meanwhile, in the EMEM model, no effect of the cytokine exposure was observed.

#### 3.4.3 Signaling Lipid Levels Differ Between the Channels

Under SF‐EMEM conditions, 18 signaling lipids remained unaffected by the cytokine exposure, but their levels in the top channel differed from those in the middle and bottom channels (Figure S5). Few bile acids and 5‐iPF2α‐VI were higher in the top channel compared to the middle and bottom channels, whereas the opposite was observed for the three eicosanoids, three docosanoids, and six long‐chain fatty acids (Figure S5). Differences in the levels of the signaling lipids between the channels, irrespective of the cytokine concentration, were also observed under EMEM conditions for 8,9‐DiHETrE, Δ17,6‐keto PGF1α, and 5‐iPF2α‐VI (Figure S6). Under those conditions, similar changes were also observed for other signaling lipids that did not meet our quality control criteria (Figure S7). Table 1 summarizes the key experimental findings across all measured parameters.

**TABLE 1:** Summary of results.

Parameter	Effect of cytokine exposure	Mechanistic explanation	SF‐EMEM vs. EMEM
TEER (Barrier integrity)	↓ TEER (increased permeability)	Cytokines disrupt tight junctions, reducing resistance	No major difference in TEER reduction
Viability (DRAQ7)	↑ DRAQ7 (increased cell death)	Cytokine exposure compromises plasma membrane integrity, allowing DRAQ7 to enter and stain the nucleus.	Similar DRAQ7 increase in both media
Actin remodeling	↑ Cytoskeletal changes	Cytokines trigger cytoskeletal remodeling via NF‐κB, MAPK	Stronger actin remodeling in SF‐EMEM
PGs and derivatives	↑ Pro‐inflammatory lipid mediators in top channel	COX‐2 upregulation leads to increased prostaglandin synthesis	Prostaglandins more prominent in SF‐EMEM
Eicosanoids (8,9‐DiHETrE, 11,12‐DiHETrE)	↓ Eicosanoids in bottom channel for some cytokine concentrations	—	Effects seen in SF‐EMEM, not as clear in EMEM

## 4 Discussion

This study uses a gut‐on‐chip model to examine the effect of inflammatory cytokines on barrier function and lipid signaling in intestinal epithelium. Our findings demonstrate that cytokine exposure not only induces significant barrier dysfunction but also triggers distinct lipid signaling responses that are influenced by both medium composition and spatial location.

Cytokine exposure consistently compromised barrier integrity, as evidenced by decreased TEER and increased DRAQ7 signal. These effects align with established mechanisms whereby TNF‐α, IL‐1β, and IFN‐γ disrupt tight junctions through NF‐κB and MAPK signaling pathways [7, 32]. The observed barrier dysfunction was accompanied by significant actin remodeling, reflecting early cytoskeletal reorganization that typically precedes junction breakdown [8].

While TEER and DRAQ7 signal changes were comparable between media conditions, actin remodeling showed medium‐dependent differences, with SF‐EMEM generally displaying lower values compared to EMEM for most parameters at specific cytokine concentrations.

Serum factors may influence cytoskeletal reorganization, likely through integrin‐mediated or growth factor‐dependent pathways [33], without directly affecting barrier integrity. The use of serum‐free conditions can standardize responses through cell cycle synchronization [25]. Although autophagy‐induced mechanisms can enhance barrier function under serum‐free conditions [34, 35], our results indicate that these effects did not measurably impact TEER or DRAQ7 signal in this model. Future studies should investigate the specific mechanisms by which serum components influence cytoskeletal responses without affecting barrier integrity measures.

A novel aspect of our study was the comprehensive profiling of lipid mediators in a gut microphysiological system, revealing distinct spatial patterns and medium‐dependent responses. In this work, a concentration‐dependent increase in nine prostaglandins and derivatives was observed in the top channel (apical side) under SF‐EMEM conditions. These findings align with other in vitro studies reporting PGE2 association with impaired barrier integrity [11, 12] and its production by Caco‐2 cells following exposure to proinflammatory cytokines IL‐1b, TNFα, and IFN‐γ [36]. Moreover, elevated levels of PGE2 have been observed in mucosal biopsies from patients with ulcerative colitis [14, 37]. Under the studied conditions, the PGs are most likely produced by cyclooxygenase‐2 (COX‐2), which in the gastrointestinal tract are upregulated in case of inflammation, instead of COX‐1 which produces PGs under physiological conditions [38, 39, 40]. While PGs increased, thromboxane B2 decreased, suggesting a shift in the COX pathway favoring prostaglandin rather than thromboxane formation. The levels of bile acids TDCA and LCA were also affected by cytokine exposure, but in opposing directions. These changes may reflect altered activation of Farnesoid X Receptor (FXR), which is increasingly recognized for its involvement in gut immunity and barrier function [41].

In the top channel, unlike under SF‐EMEM conditions, where overall PG upregulation was observed, under EMEM conditions, only PGF1α and 8‐iso‐PGF2α showed cytokine‐induced increases. Similarly, the bile acid response to the cytokine exposure also varied between the media conditions. Other signaling lipids also appeared affected under the EMEM conditions; however, the high background signal compromised the data quality. These findings suggest that media conditions influence lipid signaling and that the discrepancy in response between the media conditions likely stems from a combination of biological differences and methodological constraints.

Under neither media conditions were the results in the top channel replicated in the bottom and medium channels. While no alterations were observed under EMEM conditions in the bottom channel, a concentration‐dependent decline in eicosanoids was detected under SF‐EMEM conditions. Even though the decline is not consistent across the cytokine concentrations, the observation supports the shift of the arachidonic acid metabolism toward prostaglandin synthesis, observed in the top channel under the same conditions.

Spatial organization in the OrganoPlate was also evident by the difference in the levels of signaling lipids, unaffected by the cytokine exposure, between the top and bottom channels. Such differences were observed under both media conditions, with clearer trends observed under SF‐EMEM conditions, partly due to the high EMEM background signals. These findings demonstrate that lipid profiles can differ markedly between apical and basolateral compartments, emphasizing the importance of sampling location when interpreting epithelial lipid signaling. Future studies should explore whether these compartment‐specific patterns reflect physiologically relevant lipid trafficking processes, such as directed secretion toward the lumen or circulation, particularly under inflammatory conditions.

A key strength of our study is its multiparametric approach, integrating TEER, viability (DRAQ7), actin remodeling, and lipid signaling to provide a comprehensive view of cytokine‐induced barrier dysfunction. Real‐time TEER monitoring offers non‐invasive tracking, while lipid profiling adds a novel lipidomic dimension, linking cytokine exposure to inflammation and shifts relevant to inflammatory diseases like IBD. Actin remodeling complements these endpoints by capturing early cytoskeletal changes that contribute to tight junction disassembly and epithelial deformation, which are key features of barrier loss in IBD [8].

Several oxylipin species, including eicosanoids, have increasingly been recognized in mediating inflammatory responses in IBD [14, 42, 43]. By following these lipid mediators in our gut‐on‐chip model, we demonstrate that this system captures clinically relevant inflammatory pathways, making it suitable for studying IBD‐related mechanisms and screening therapeutic interventions targeting lipid signaling cascades.

Unlike previous studies in the field that focused on a narrow range of eicosanoids [11, 12, 36], this work examined a broader spectrum of eicosanoids and other signaling lipids. The study also systematically compares serum‐containing (EMEM) and serum‐free (SF‐EMEM) media, highlighting how medium composition affects inflammation‐related lipid signaling. These findings emphasize the importance of standardized culture conditions when studying gut inflammation. Together, these strengths establish this study as a proof‐of‐concept for integrating gut barrier function and lipid signaling in inflammation research.

Several limitations of this study should be considered. First, our model examines acute inflammation over 72 h and does not fully capture the chronic inflammatory conditions characteristic of many intestinal diseases [44]. Second, while the Caco‐2 monoculture provides reproducible results, it lacks immune cells, microbiota, and a mucus layer, all of which play critical roles in intestinal barrier function and inflammatory responses [30, 45, 46]. Technical aspects of lipid analysis required pooling multiple chips for adequate sample volume, which reduced the number of replicates. Subsequent studies should therefore aim to develop analytical assays with enhanced sensitivity. The distinct spatial distribution of lipids between compartments and their varying responses to cytokine treatment suggest complex transport dynamics that warrant further investigation.

Important to note is that although cytokine exposure consistently triggered changes in DRAQ7 staining, actin remodeling, and prostaglandin secretion, this study does not establish causal relationships between these outcomes. These endpoints should therefore be interpreted as parallel responses to inflammatory stress. Future studies will be needed to determine whether specific lipid mediators actively drive epithelial structural or functional changes.

Our study provides a comprehensive analysis of cytokine‐induced intestinal barrier dysfunction and lipid signaling using a gut‐on‐chip model. By integrating TEER, DRAQ7 staining, actin remodeling, and lipid profiling, we reveal distinct inflammatory responses influenced by medium composition and spatial organization. Our findings demonstrate that cytokine exposure consistently impairs barrier integrity while inducing prostaglandin‐mediated lipid signaling, with notable differences between serum‐free and complete media conditions.

While this proof‐of‐principle study establishes a valuable framework for investigating gut inflammation, future research should extend to chronic inflammation models and refine lipidomic analysis. Follow‐up experiments directly exposing gut epithelial tubules to selected prostaglandins could help establish causal links between specific lipid mediators and functional barrier responses, such as TEER reduction, viability, and actin remodeling. Future work could also explore the lipid signaling response to individual cytokines to clarify their specific contributions. Incorporating other cell types such as immune‐ or mucus‐producing cells would further elucidate how these lipid signals mediate immune‐epithelial crosstalk during inflammation. This could inform the development of targeted combination therapies addressing multiple inflammatory nodes simultaneously—particularly valuable for complex conditions like IBD.

## Author Contributions

**M.M**.: conceptualization; formal analysis; investigation; methodology; visualization; writing, original draft; writing, review, and editing; **M.V.S**.: conceptualization; formal analysis; investigation; methodology; visualization; writing, original draft; writing, review and editing; **K.Q**.: conceptualization; funding acquisition; resources; supervision; writing, review and editing; **A.C.H**.: conceptualization; resources; supervision; writing, review and editing; **T.H**.: conceptualization; funding acquisition; project administration; resources; supervision; writing, review and editing.

## Conflicts of Interest

All authors have read the journal's policy on disclosure of potential conflicts of interest. K.Q. and M.M. are employees of Mimetas BV, which markets advanced in vitro systems for drug development. T.H. is a shareholder of the same company. The authors declare they have no additional conflicts of interest.

## Supporting information


**Data S1:** Supplementary Information.

**Table S1:** TEER statistical analysis.
**Table S2:** DRAQ7 statistical analysis.
**Table S3:** Actin count statistical analysis.
**Table S4:** Actin total area statistical analysis.
**Table S5:** Actin object area statistical analysis.
**Table S6:** List of the signaling lipids that passed the quality control.

## Data Availability

Included in article.
