# Supplementary material for: Cytokine‐Induced Barrier Dysfunction and Lipid Signaling in a Gut‐On‐Chip Model
Source: FASEB J. 2025 Oct 9;39(19):e71059. doi: 10.1096/fj.202501685R (PMC12510296; doi:10.1096/fj.202501685R)
Supplement: Supplementary file 1 — Data S1: Supplementary Information. [file FSB2-39-e71059-s002.docx]

**Supplemental material**

**Cytokine-induced barrier dysfunction and lipid signaling in a gut-on-chip model**

Moran Morelli^1^*, Mariyana V. Savova^2^*, Karla Queiroz^1^, Amy C. Harms^2,#^, Thomas Hankemeier^2,#^

^1^Mimetas, Oegstgeest, the Netherlands

^2^Metabolomics and Analytics Center, Leiden Academic Centre for Drug Research, Leiden University, the Netherlands

*Moran Morelli and Mariyana V. Savova should be considered joint first authors

#joint corresponding authors: dr. Amy C. Harms and Prof. dr. T. Hankemeier, Metabolomics and Analytics Centre, Leiden Academic Centre for Drug Research, Leiden University, Leiden 2333 CC, Netherlands

E-mail:  a.c.harms@lacdr.leidenuniv.nl and hankemeier@lacdr.leidenuniv.nl, Tel: +31 715274226

**List of reagents for cellular assays**

| **Reagent** | **Supplier** | **Reference number** | **Note** |
| --- | --- | --- | --- |
| ActinGreen™ 488 ReadyProbes™ Reagent | Sigma-Aldrich | R37110 | 2 drops/ml |
| NucBlue™ Live ReadyProbes™ Reagent | Sigma-Aldrich | R37605 | 2 drops/ml |
| DRAQ7™ Dye | Biostatus | DR71000 | Working: 3 µM |
| Triton 100X | Sigma-Aldrich | T8787 |  |
| Formaldehyde | Sigma-Aldrich | 252549 |  |
| HBSS | ThermoFisher | 14025-092 |  |
| PBS | Gibco | 70013-016 | Diluted 10X in milliQ |
| EMEM | ATCC | 30-2003 | Complete EMEM:  440mL EMEM  50mL FBS  5mL P/S  5mL NEAA  SF-EMEM  490mL EMEM  5mL P/S  5mL NEAA |
| FBS | Gibco | 16140-071 |  |
| P/S | Gibco | 15140-122 |  |
| MEN NEAA | Gibco | 11140-050 |  |
| OrganoReady Colon Caco-2 3-lane 40 | MIMETAS | MI-OR-CC-01 |  |
| TNF-α | Immunotools | 11343015 | 50ug in 500uL water to get 100ug/mL, then 1:500 in culture medium |
| IL-1β | Immunotools | 11340015 | 50ug in 500uL water to get 100ug/mL, then 1:500 in culture medium |
| IFN-γ | Preprotech | 300-02 | 20ug in 200uL water to get 100ug/mL, then 1:500 in culture medium |

**Chemicals and reagents for lipid profiling**

LC-MS grade acetonitrile, methanol, isopropanol, glacial acetic acid were acquired from Biosolve (Valkenswaard, Netherlands); 1-Butanol (99%) from Thermo Scientific (Belgium); Butylated hydroxytoluene (≥99%), ethylenediaminetetraacetic acid (≥99%) from Sigma-Aldrich; citric acid monohydrate was purchased from Roth (Karlsruhe, Germany); disodium hydrogen phosphate (≥99.5, Supelco, Merck, Darmstadt, Germany). Purified MilliQ water was obtained from Arium® pro VF system (Sartorius Stedim biotech, Göttingen, Germany). Isotopicaly labelled standards were purchased from Cayman Chemicals (Ann Arbor, MI, USA) and Avanti Polar Lipids (Alabaster, AL, USA) as previously described.^1^


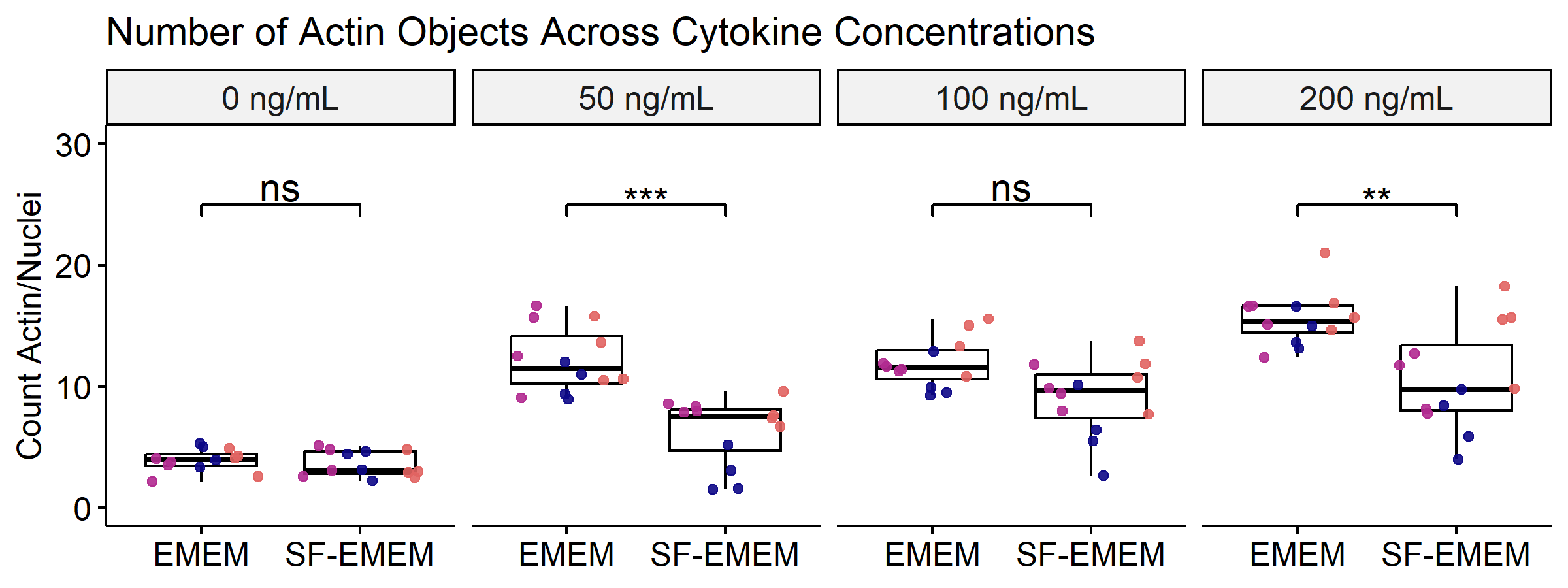


**Figure S1 Number of actin objects across cytokine concentrations in EMEM and SF-EMEM media.** Boxplots show the count of actin objects per nucleus in Caco-2 tubules exposed to increasing cytokine concentrations (0, 50, 100, and 200 ng/mL) in EMEM and SF-EMEM. Each dot represents a measurement from a single chip in the OrganoPlate. Colors indicate independent experiments. Results are derived from three independent experiments (N = 3), with four technical replicates per cytokine concentration (n = 4). Statistical significance between media types at each cytokine concentration was determined using an unpaired t-test, with p-values adjusted for multiple comparisons using the Bonferroni correction. Significance levels are indicated as follows: *** p<0.001, * p<0.05, ns: not significant.


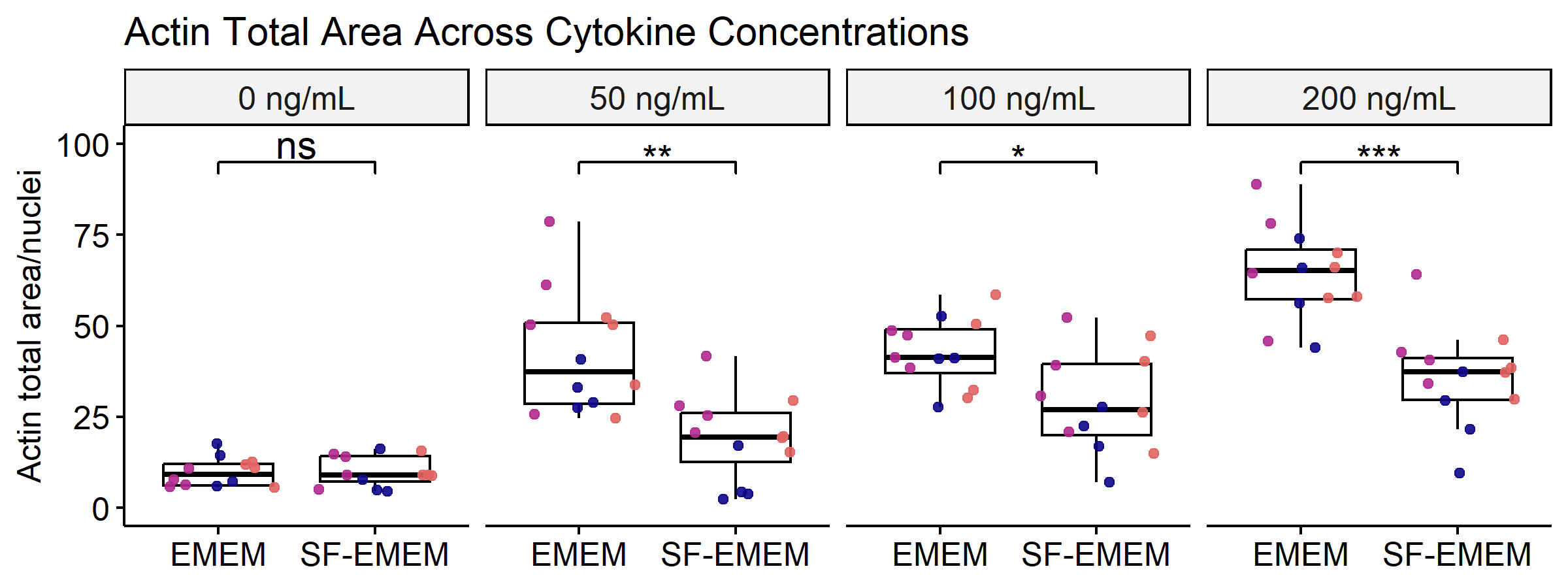


**Figure S2 Total area of actin objects across cytokine concentrations in EMEM and SF-EMEM media.** Boxplots show the total area of actin objects per nucleus in Caco-2 tubules exposed to increasing cytokine concentrations (0, 50, 100, and 200 ng/mL) in EMEM and SF-EMEM. Each dot represents a measurement from a single chip in the OrganoPlate. Colors indicate independent experiments. Results are derived from three independent experiments (N = 3), with four technical replicates per cytokine concentration (n = 4). Statistical significance between media types at each cytokine concentration was determined using an unpaired t-test, with p-values adjusted for multiple comparisons using the Bonferroni correction. Significance levels are indicated as follows: *** p<0.001, ** p<0.01, * p<0.05, ns: not significant.


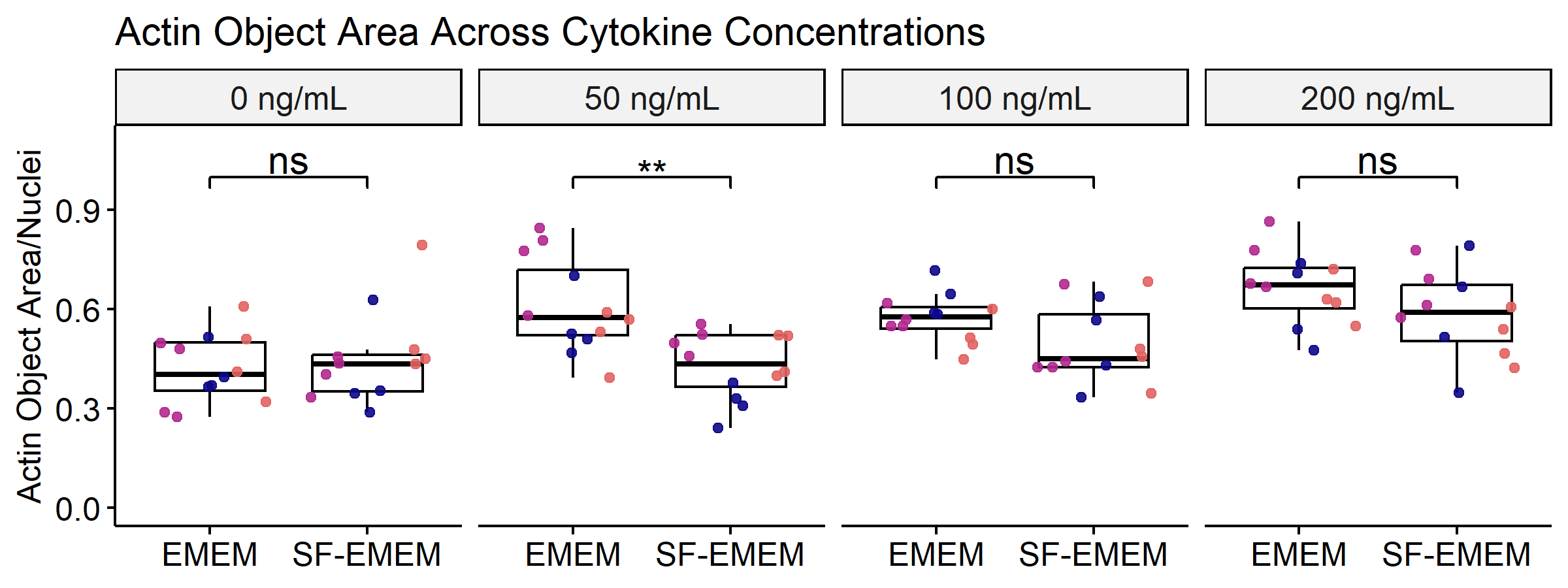


**Figure S3 Average area of actin objects across cytokine concentrations in EMEM and SF-EMEM media.** Boxplots show the area of actin objects per nucleus in Caco-2 tubules exposed to increasing cytokine concentrations (0, 50, 100, and 200 ng/mL) in EMEM and SF-EMEM. Each dot represents a measurement from a single chip in the OrganoPlate. Colors indicate independent experiments. Results are derived from three independent experiments (N = 3), with four technical replicates per cytokine concentration (n = 4). Statistical significance between media types at each cytokine concentration was determined using an unpaired t-test, with p-values adjusted for multiple comparisons using the Bonferroni correction. Significance levels are indicated as follows: *** p<0.001, ** p<0.01, * p<0.05, ns: not significant.


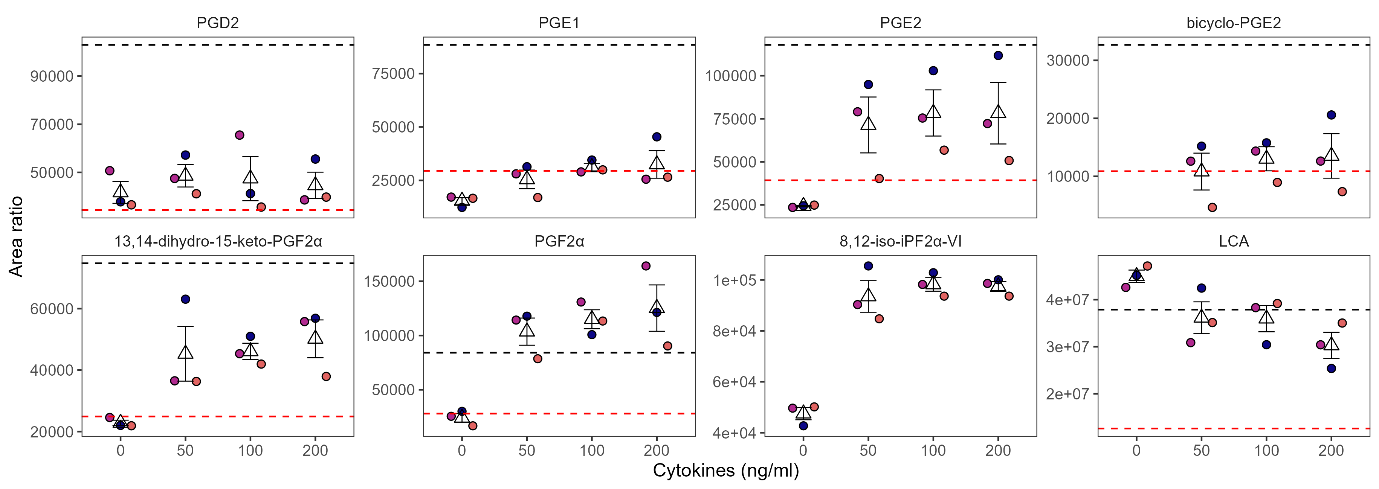


**Figure S4 Impact of cytokine exposure on signaling lipids in EMEM media, apical side that did not meet the quality control criteria (Table S2).** Results are derived from three independent experiments (N = 3). Dot colors indicate independent experiments. The triangular marker represents the mean of the three replicates, while the error bars are based on the standard deviation. The red and black dotted lines represent respectively one and three times the median level in blank SF-EMEM.


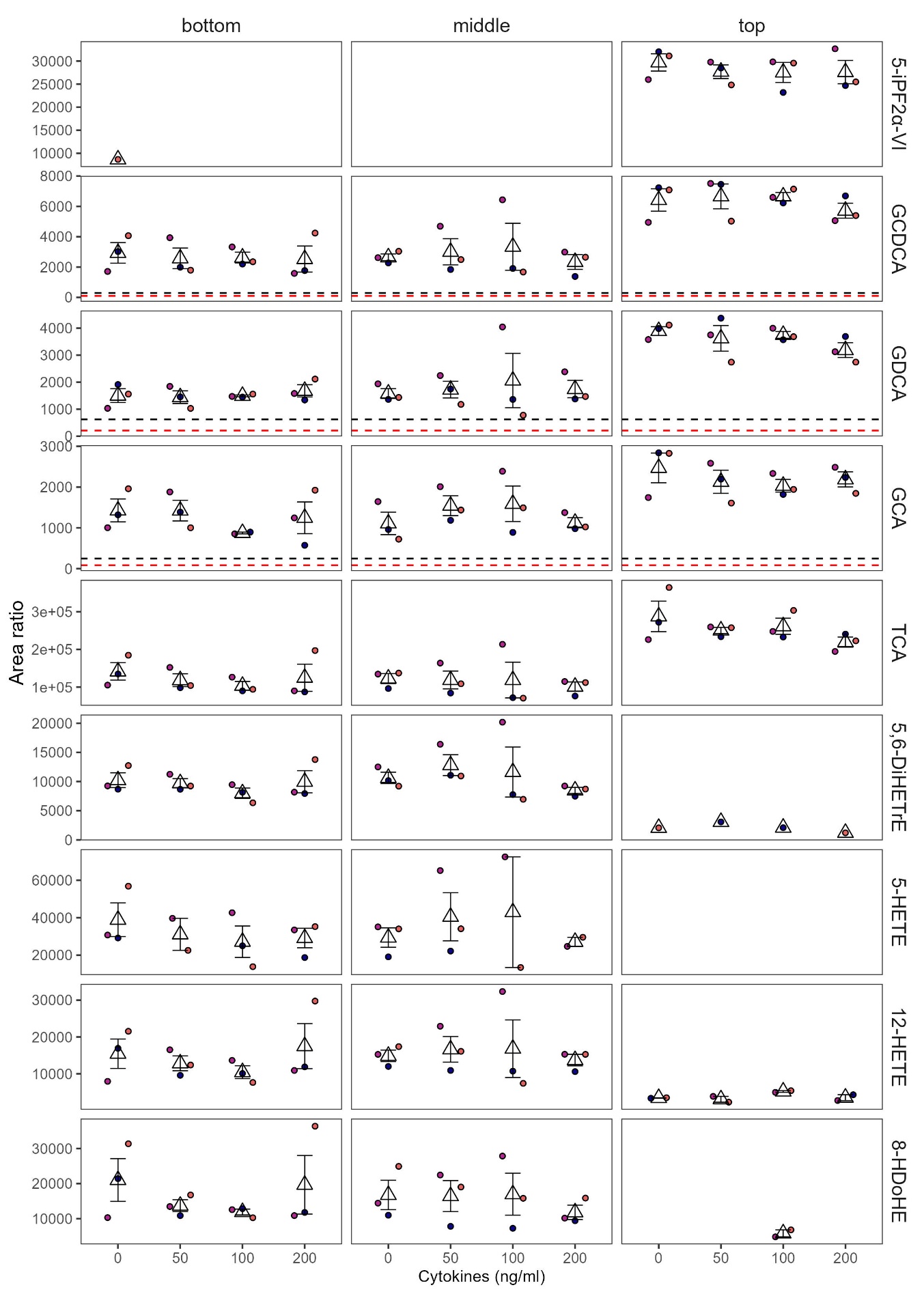


**Figure S5. Signaling lipids with levels, irrespective of the cytokine concentration, that are lower or higher in the top channel, compared to the middle and bottom channels under SF-EMEM media conditions.** Results are derived from three independent experiments (N = 3) except for 5-iPF2α-VI in the bottom channel where only 1 measurement was above the detection limit. Dot colors indicate independent experiments. The triangular marker represents the mean of the three replicates, while the error bars are based on the standard deviation. The red and black dotted lines represent respectively one and three times the median level in blank SF-EMEM.


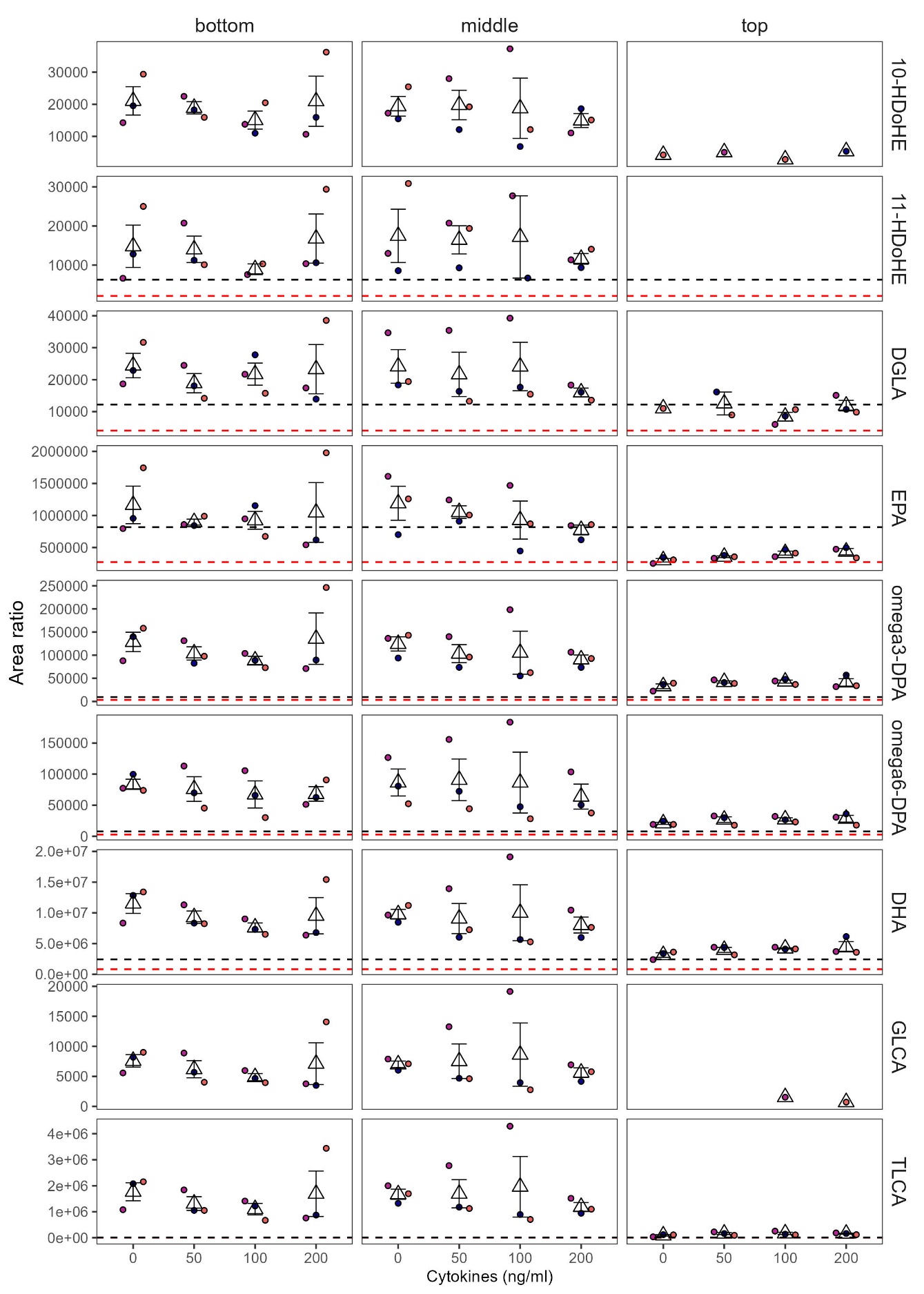


**Figure S5 (continued). Signaling lipids with levels, irrespective of the cytokine concentration, that are lower or higher in the top channel, compared to the middle and bottom channels under SF-EMEM media conditions.** Results are derived from three independent experiments (N = 3). Missing measurement points correspond to signals below the detection limit. Dot colors indicate independent experiments. The triangular marker represents the mean of the three replicates, while the error bars are based on the standard deviation. The red and black dotted lines represent respectively one and three times the median level in blank SF-EMEM.


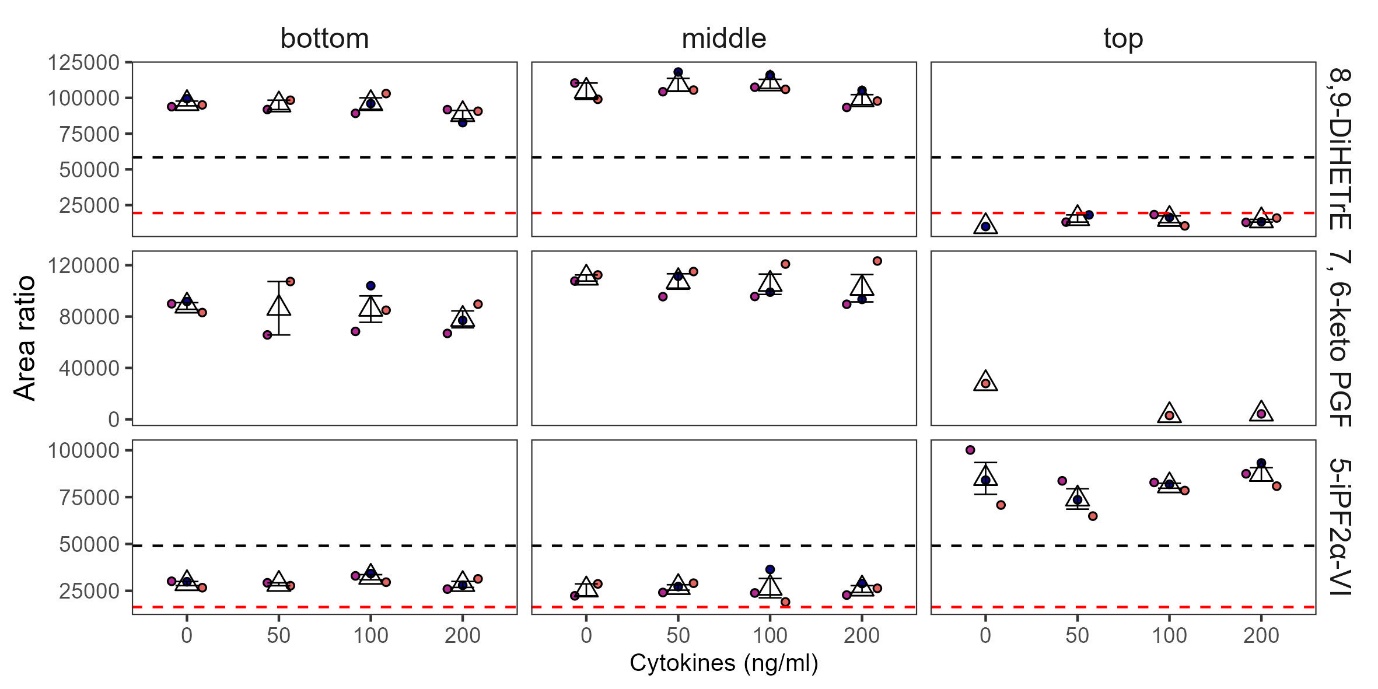


**Figure S6. Signaling lipids with levels, irrespective of the cytokine concentration, that are lower or higher in the top channel, compared to the middle and bottom channels under EMEM media conditions.** Results are derived from three independent experiments (N = 3) except measurements of bottom channel 50 ng/ml and middle channel 0 ng/ml where the replicates were 2. Other missing measurement points correspond to signals below the detection limit. Dot colors indicate independent experiments. The triangular marker represents the mean of the three replicates, while the error bars are based on the standard deviation. The red and black dotted lines represent respectively one and three times the median level in blank EMEM.


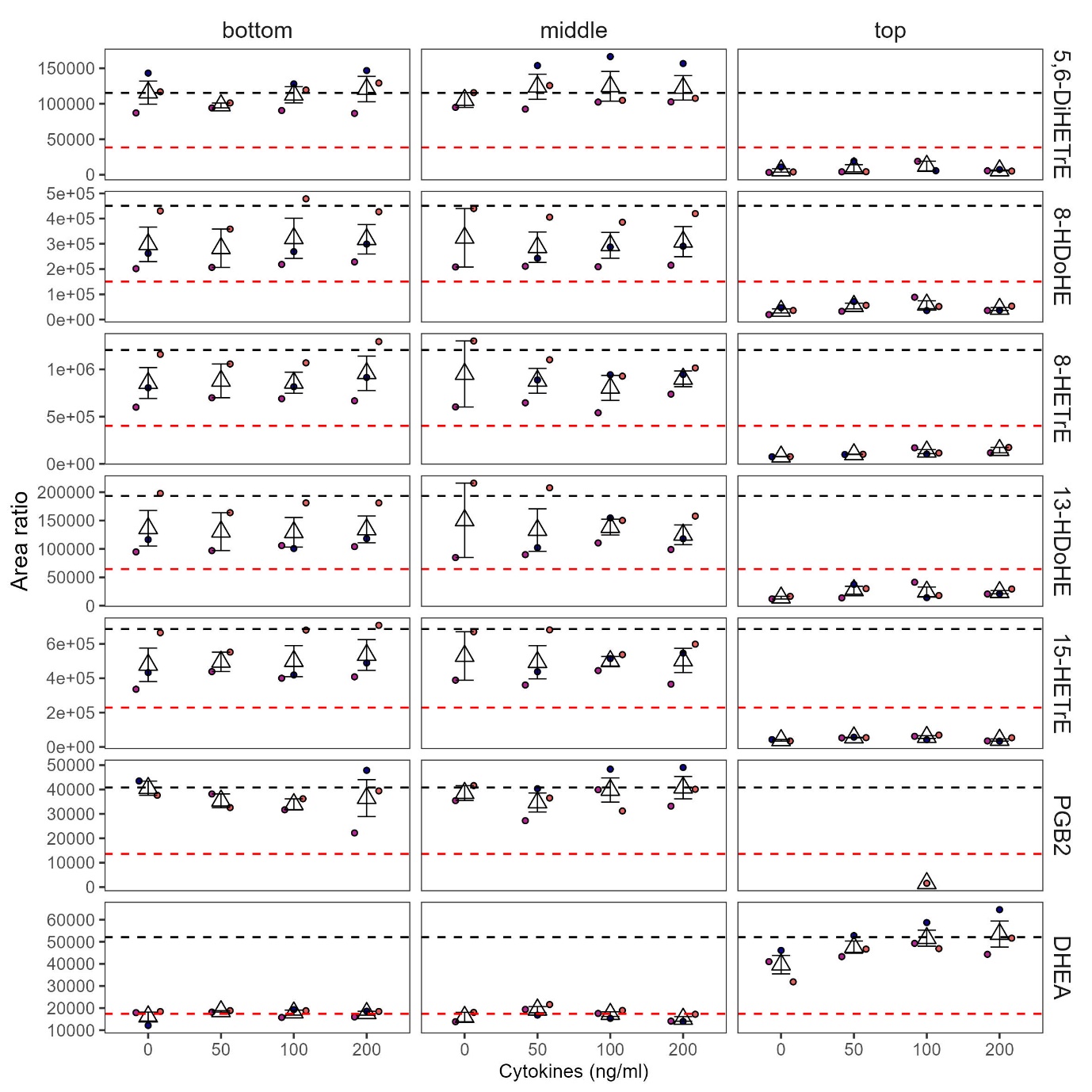


**Figure S7. Fraction of the signaling lipids with levels, irrespective of the cytokine concentration, that are lower or higher in the top channel, compared to the middle and bottom channels under EMEM media conditions but did not meet out quality control.** Results are derived from three independent experiments (N = 3) except measurements of bottom channel 50 ng/ml and middle channel 0 ng/ml where the replicates were 2. Other missing measurement points correspond to signals below the detection limit. Dot colors indicate independent experiments. The triangular marker represents the mean of the three replicates, while the error bars are based on the standard deviation. The red and black dotted lines represent respectively one and three times the median level in blank EMEM.

**References**

1. Yang, W. *et al.* A comprehensive UHPLC-MS/MS method for metabolomics profiling of signaling lipids: Markers of oxidative stress, immunity and inflammation. *Anal Chim Acta* **1297**, 342348 (2024).
